# Two-Step Delivery: Exploiting the Partition Coefficient Concept to Increase Intratumoral Paclitaxel Concentrations *In vivo* Using Responsive Nanoparticles

**DOI:** 10.1038/srep18720

**Published:** 2016-01-07

**Authors:** Aaron H. Colby, Rong Liu, Morgan D. Schulz, Robert F. Padera, Yolonda L. Colson, Mark W. Grinstaff

**Affiliations:** 1 Departments of Biomedical Engineering and Chemistry, Metcalf Center for Science and Engineering, Boston University, Boston, MA 02215; 2 Division of Thoracic Surgery, Department of Surgery, Brigham and Women’s Hospital, Boston, MA 02115; 3 Department of Pathology, Brigham and Women’s Hospital, Boston, MA 02115.

## Abstract

Drug dose, high local target tissue concentration, and prolonged duration of exposure are essential criteria in achieving optimal drug performance. However, systemically delivered drugs often fail to effectively address these factors with only fractions of the injected dose reaching the target tissue. This is especially evident in the treatment of peritoneal cancers, including mesothelioma, ovarian, and pancreatic cancer, which regularly employ regimens of intravenous and/or intraperitoneal chemotherapy (e.g., gemcitabine, cisplatin, pemetrexed, and paclitaxel) with limited results. Here, we show that a “two-step” nanoparticle (NP) delivery system may address this limitation. This two-step approach involves the *separate* administration of NP and drug where, first, the NP localizes to tumor. Second, subsequent administration of drug then rapidly concentrates into the NP already stationed within the target tissue. This two-step method results in a greater than 5-fold increase in intratumoral drug concentrations compared to conventional “drug-alone” administration. These results suggest that this unique two-step delivery may provide a novel method for increasing drug concentrations in target tissues.

The distribution or partition of an organic compound between two phases is a fundamental occurrence observed in the chemical and physical sciences, with significant practical implications across numerous industries including petroleum, food, cosmetic, and pharmaceutical. In the pharmaceutical arena, drug dose, high local target tissue concentration, and prolonged duration of exposure are essential criteria in achieving optimal drug performance^1^,^2^,^3^,^4^,^5^,^6^. However, systemically delivered drugs often fail to effectively address these factors with only fractions of the injected dose reaching the target tissue. This is especially evident in the treatment of peritoneal cancers, including mesothelioma, ovarian, and pancreatic cancer, which regularly employ regimens of intravenous and/orintraperitoneal chemotherapy (e.g., gemcitabine, cisplatin, pemetrexed, and paclitaxel) with limited results^7^,^8^,^9^. We hypothesized that a polymeric, crosslinked-network located within a tumor and which partitions a drug from an aqueous solution could be used as a drug-concentrating device to increase overall tumoral drug levels *in vivo*.

In this study, we use a 100 nm pH-responsive expansile NP (eNP) composed of a cross-linked polymer network that, upon cellular internalization and exposure to the mildly acidic (pH ~5) endosome, expands to afford a 1000 nm gel particle (Fig. 1, right)^10^,^11^. eNPs are synthesized using a previously reported, mini-emulsion, polymerization technique with the covalent incorporation of a fluorophore (PolyFluor 570®; PolyFluor® 407; or pyrene,) to enable subsequent visualization or characterization studies^10^,^11^,^12^. Here, we describe a “two-step” delivery system which tests this hypothesis and addresses the above limitation. This two-step approach involves the *separate* administration of nanoparticles and drug where: first, the nanoparticles localize to tumor; and, second, subsequent administration of drug then rapidly partitions and concentrates into the nanoparticle already stationed within the target tissue (Fig. 1, left). Specifically, using paclitaxel (Pax), a responsive polymeric nanoparticle that swells to afford a crosslinked network at mildly acidic pH (expansile nanoparticles, eNPs; Fig. 1, right^10^,^11^), covalent incorporation of fluorophores to enable subsequent visualization or characterization of eNPs^10^,^11^,^12^, a human-derived mesothelioma cell line (MSTO-211H), and an *in vivo* murine model of human peritoneal mesothelioma, we report: 1) the partitioning of Pax into swollen expansile nanoparticles or gel particles, and the importance of the particle composition on Pax partitioning; 2) the use of unloaded-eNPs to concentrate separately administered Pax *in vitro*; 3) the localization of fluorescently labeled eNPs to established *in vivo* mesothelioma tumors and subsequent co-localization of separately administered fluorescently labeled Pax; and, 4) the quantification of tumor tissue concentrations of Pax when administered as eNPs + Pax, Pax alone, encapsulated Pax (Pax-eNP), and poly(lactic-*co*-glycolic) acid (PLGA)-NPs + Pax as a generic, non-responsive NP control.

Paclitaxel is one of several chemotherapeutic agents used in the treatment of peritoneal carcinomatoses. However, 50% of an intraperitoneally (IP) injected dose of Pax is cleared within 3 hours of administration affording only minimal drug concentration in the tumor and short drug-tumor exposure times^13^. Overcoming this pharmacokinetic pattern of short drug exposure times and low drug concentrations presents a significant challenge to optimizing Pax delivery and efficacy. First, Pax is a cell cycle specific drug that acts only during cell replication; with only 10–15% of tumor cells expected to be in mitosis at any given time, tumoral response to Pax is reduced with short exposure times^14^,^15^,^16^,^17^. Second, the mechanism of Pax antitumor activity is dependent on Pax concentration; when tumor cells are exposed to low concentrations of Pax, the cells enter a resting state where Pax is no longer effective in causing cell death/apoptosis^18^,^19^,^20^,^21^. Increasing Pax concentration directly within the tumor tissue is, therefore, a key challenge in oncology.

## Results and Discussion

To characterize the ability of a swollen eNP, or gel particle, to sequester Pax from aqueous solution we performed a dialysis partitioning experiment according to a previously published protocol^22^. The results demonstrated that Pax partitions into the gel particle in a >4:1 ratio relative to the surrounding aqueous sink (i.e., the concentration of Pax within the eNP >4X the concentration of Pax within the surrounding aqueous environment; Fig. 2a). We suspect this result is a consequence of the chemical structure of the gel particle and the increased hydrophobicity of the gel particle compared to the aqueous phase. Synthesis and subsequent study of a similar eNP that, upon swelling, affords two succinic acid moieties instead of two hydroxyls, resulted in significantly less (i.e., 2-fold) partitioning of Pax into the gel particle phase (Fig. 2a). To estimate the relative hydrophobicity of the gel particle, we used the solvochromatic dye, pyrene, as a polarity indicator following a published procedure^11^. As shown in Fig. 2b, swollen gel-like pyrene-labeled-eNPs (i.e., at pH 5) possess a polarity index similar to ethyl acetate and lower than water, indicating the relatively hydrophobic environment of the gel particles. This finding is important because the partition coefficient for Pax in ethyl acetate is the highest from aqueous solution compared to other common organic solvents^23^ and, thus, provides the motivation for the proposed two-step eNP-mediated delivery of Pax.

An initial *in vitro* study was performed to confirm that eNPs would concentrate Pax into mesothelioma cells after a 48 hr co-incubation period in the presence of media containing serum (Fig. 3a). For these studies, MSTO-211H human mesothelioma tumor cells were cultured under standard conditions prior to a “pre-treatment” co-incubation period of 48 hr with media alone or with one of two unloaded nanoparticle formulations (neither containing Pax): eNPs or PLGA-NPs (generic NP control). Paclitaxel labeled with Oregon Green (green-Pax) was then added to all cultures for 4 hr. Cells were washed 3X with phosphate buffered saline (PBS) to remove green-Pax adsorbed to the cell surface. Confocal microscopy confirmed the intracellular accumulation of green-Pax and demonstrated co-localization with PolyFluor 570®-labeled-eNPs (red-eNPs) in red-eNP pre-treated cells (Fig. 3b). Due to the low concentration of green-Pax used and the short time of incubation (4 hr), no green-Pax was detectable by confocal microscopy in the control cells. To quantify internalized green-Pax, cells were washed, lysed and then the internalized green-Pax was measured with a fluorescent plate reader. Intracellular accumulation of green-Pax was nearly 5X greater in cells pre-treated with eNPs than in cells pre-treated with media or PLGA-NPs (Fig. 3c). Importantly, the partitioning of Pax into the eNP does not eliminate the drug’s cytotoxic affect as demonstrated by the significant improvement in efficacy of paclitaxel-loaded-eNPs (i.e., Pax-eNPs) compared to Pax alone treatments indicating that Pax is released from eNPs and is able to exert its antitumoral activity^10^,^12^,^24^.

We next characterized the ability of eNPs to concentrate green-Pax into a tumor *in vivo*. Despite the absence of tumor-specific targeting ligands, previously published studies in murine models of established IP mesothelioma demonstrated that eNPs readily localize to areas of tumor *in vivo*^12^. Leveraging this eNP localization, we hypothesized that eNPs could be utilized to increase tumor tissue concentrations of separately administered Pax using a two-step approach: 1) an initial dose of unloaded eNPs that localizes to tumor; and, 2) a second, separately injected dose of Pax which then concentrates within the tumor-associated eNPs.

In order to simultaneously visualize the location of both eNPs and drug *in situ*, we fluorescently labeled eNPs with PolyFluor® 407 (i.e., blue-eNPs) and used Oregon-Green labeled Pax (i.e., green-Pax). Selection of these two fluorophores allows separate visualization of the PolyFluor® 407 (i.e., eNP) and Oregon Green (i.e., Pax) using 254 nm and 365 nm wavelength UV light, respectively. This fluorophore-based tracking technique was chosen for its ease-of-use and accessibility (handheld Wood’s lamps provide the requisite UVC and UVA emission wavelengths) with the knowledge that full biodistribution studies would be conducted in the future using radiolabeled compounds.

Following the establishment of human MSTO-211H tumors for 13 days in a murine model of peritoneal mesothelioma, unloaded blue-eNPs were injected IP into three mice and allowed 48 hr to localize to the tumor tissue. Three control mice received an equivalent volume injection of saline. Forty-eight hours later, a single IP dose of 10 mg/kg green-Pax, solubilized in Cremophor/ethanol (C/E), was administered IP to all mice. Three days following green-Pax injection (sufficient time to ensure clearance of >99% of free Pax^25^), mice were sacrificed and fluorescent imaging was utilized to identify the location of blue-eNPs and green-Pax. Figure 4a displays representative images of blue-eNP vs. saline pre-treated mice immediately following sacrifice. Visible light images confirm that tumor was present in all animals and identifies the location of several large tumor nodules. In blue-eNP treated animals, imaging with 254 nm UV light shows co-localization of blue-eNPs within these tumor nodules and 365 nm UV light excitation shows further co-localization of green-Pax with both the blue-eNPs and tumor nodules. In saline pre-treated mice, no PolyFluor® 407 (i.e., blue-eNP) signal was observed and Oregon Green (i.e., green-Pax) signal was not visibly detectable, consistent with the known rapid clearance of Pax *in vivo*. These gross results were confirmed via histological sectioning of tumors from each treatment group and subsequent examination by confocal microscopy with DAPI and FITC filters to isolate the blue-eNP and green-Pax signals, respectively (Fig. 4b). As in the gross *in situ* images, confocal images show green-Pax and blue-eNPs co-localized within the tumor microenvironment while green-Pax is not present or detected in the tumor tissue of animals receiving saline pre-treatments.

**Figure 1:**
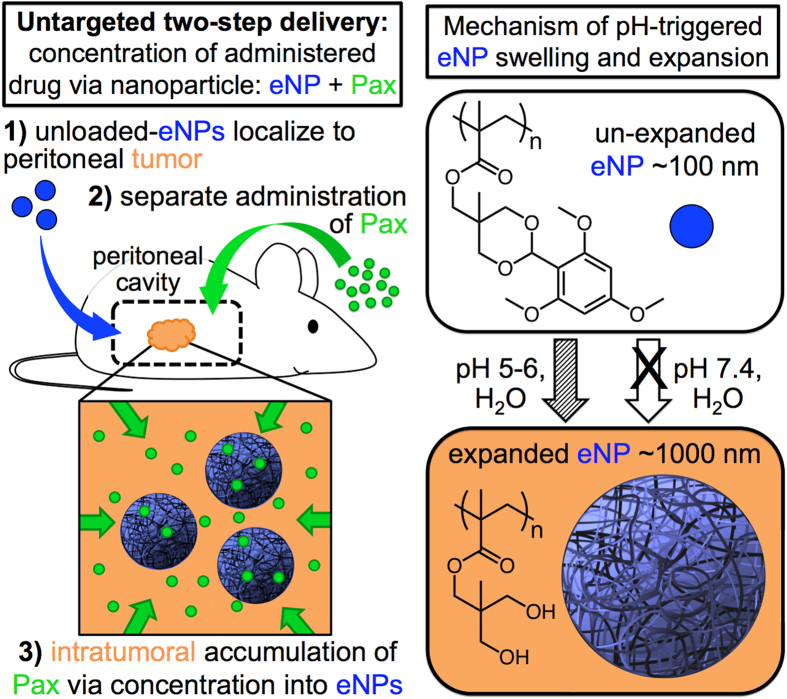
(left) Schematic illustrating the paradigm of untargeted two-step delivery via: 1) a pre-treatment of unloaded particles followed by, 2) subsequent and separate administration of the agent (e.g., paclitaxel; Pax) leading to 3) intratumoral accumulation of Pax. (**right**) Chemical structure and mechanism of particle expansion/swelling following exposure to a mildly acidic aqueous environment.

**Figure 2:**
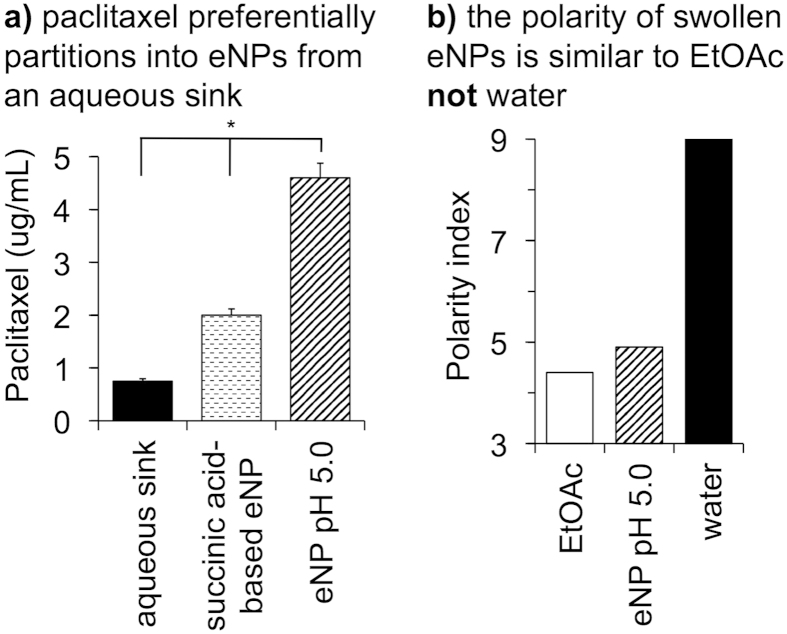
(**a**) At pH 5 the eNP, though hydrophilic enough to swell with water, still provides a significantly more hydrophobic environment for Pax than the surrounding water and results in the partitioning of Pax into the swollen eNP. A more hydrophilic eNP with succinic acid-based functionalities does not constitute as favorable an environment for Pax resulting in less accumulation of Pax within the particles. (**b**) The swollen eNP (pH 5.0) is similar in hydrophobicity/polarity to ethyl acetate (EtOAc)—an organic solvent with a high partition coefficient for Pax in a water/EtOAc mixture.

**Figure 3:**
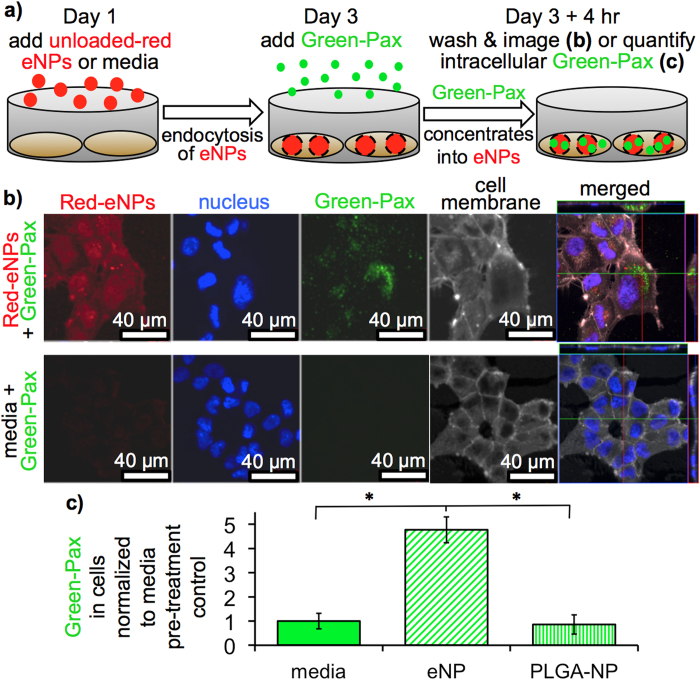
*In vitro* characterization and quantification of the drug concentrating effect. (**a**) Schematic illustration of the experimental design. (**b**) Confocal microscopy images show green-Pax internalization in cells receiving a red-eNP pre-treatment. (**c**) Green-Pax uptake quantified via a fluorescent plate reader is significantly (* *P* < 0.01) increased in cells receiving a pre-treatment of eNPs compared to cells receiving media or PLGA-NP pre-treatments.

**Figure 4:**
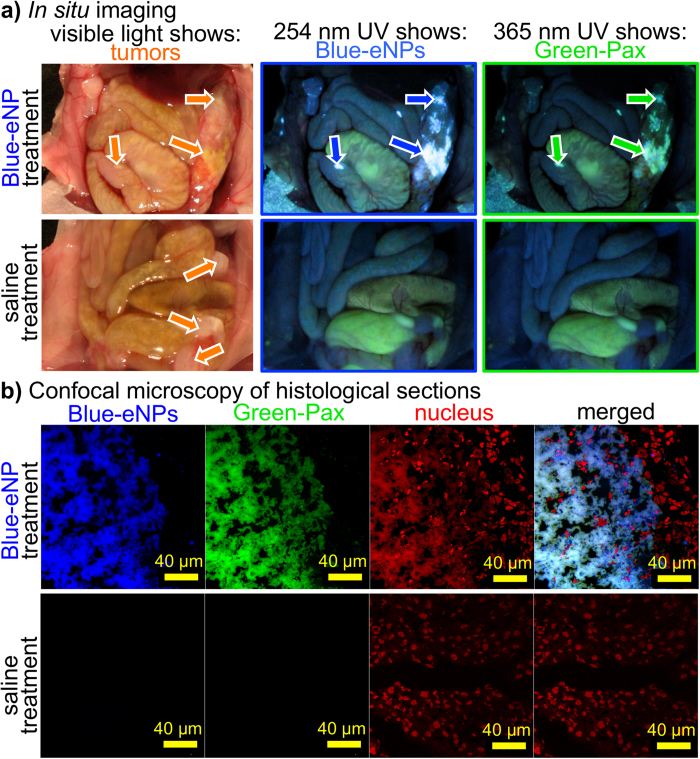
*In vivo* demonstration of the drug concentrating effect using blue-eNPs + green-Pax in a murine model of established IP mesothelioma. (**a**) In blue-eNP pre-treated animals **(top row)**, *in situ* imaging of the IP space under visible light reveals large tumor nodules **(left)**, while imaging with 254 nm light shows blue-eNPs **(middle)** and 365 nm light reveals green-Pax **(right)** co-localized within tumor tissue. When saline is the pre-treatment **(bottom row),** no blue-eNP or green-Pax signal are visible in the tumor tissue. (**b**) Confocal microscopy of these tumors confirms co-localization of blue-eNPs and green-Pax within tumor tissue (blue, blue-eNP; green, green-Pax; red, nucleus) as well as absence of both blue-eNPs and green-Pax in saline treated animals.

**Figure 5:**
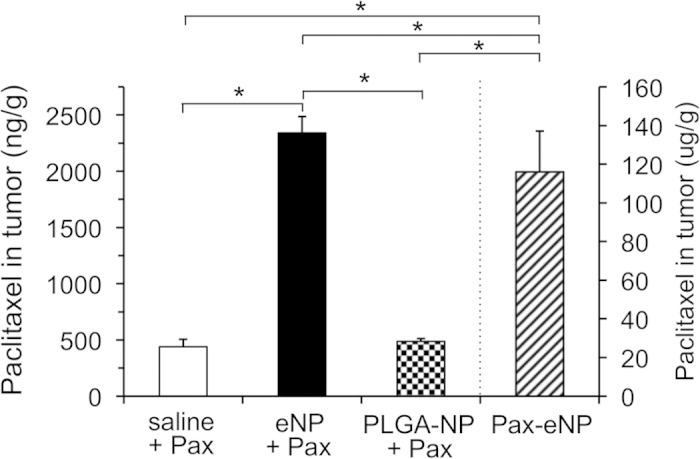
Quantification of the drug concentrating effect *in vivo* using eNPs + Pax in a murine model of established IP mesothelioma. Pax concentrations in the tumor tissue for eNP pre-treated animals **(solid black**; n = 21) are significantly higher than in animals receiving a pre-treatment of saline **(white**; n = 14) or PLGA-NPs **(thatched**; n = 13) (* *P* < 0.05). Pax concentrations are significantly higher in Pax-eNP treated (**hashed**; n = 10) animals than for all other treatments. Values are mean ± standard error.

Having confirmed qualitatively that fluorescently labeled eNPs concentrate subsequently administered green-Pax into IP tumors, we quantified this effect using unlabeled eNPs/Pax and liquid chromatography-triple quadrapole mass spectrometry. The animals were treated exactly as before, with the exception that unlabeled-eNPs and Pax were administered instead of blue-eNPs and green-Pax. Two additional treatment groups were added to this study: 1) unloaded-PLGA-NPs (i.e., a comparative generic NP control); and, 2) paclitaxel-loaded eNPs (i.e., Pax-eNPs; as a comparison to traditional drug-loaded particles). The succinic acid-based eNP formulation was not tested due to its significantly decreased ability to concentrate Pax *in vitro* compared to eNPs (Fig. 1). Upon animal sacrifice 3-days after Pax IP injection, all visible tumor burden was harvested in a blinded fashion, followed by subsequent extraction and quantification of Pax by LC/MS/MS. Pre-treatment with eNPs significantly increased intra-tumoral concentrations of Pax compared to pre-treatment with either saline or PLGA-NPs (Fig. 5). The average Pax concentration within tumors in eNP pre-treated animals (2,341 ± 335 ng/g; n = 21) was significantly higher than the concentration in saline (439 ± 125 ng/g; n = 14) and PLGA-NP (487 ± 45 ng/g; n = 13) pretreated animals (*P* < 0.05 for eNP vs. PLGA-NP and eNP vs. saline; *P* = NS for PLGA-NP vs. saline). Pax-eNPs delivered ~50-fold more drug to the tumor tissue than is achieved with eNP + Pax and ~250-fold more drug than is achieved with either saline + Pax or PLGA-NPs + Pax. Thus, in the current system, traditional encapsulation of paclitaxel results in significantly more efficient delivery than the two-step method—this result is not surprising. The Pax-eNP tumoral concentration represents the upper limit of tumoral delivery possible with the eNP + Pax system. We did not observe any toxic or adverse effects during the course of this study, and these observations are in agreement with published reports with the eNP and Pax-eNP treatment groups^10^,^12^,^26^,^27^.

Importantly, the above results lay the foundation for this two-step delivery approach and provide motivation for further studies on: 1) increasing Pax accumulation in the swollen eNP or gel particle; 2) complete quantification of the biodistribution and toxicity of both eNP and Pax using radiolabeled materials; and, 3) evaluating the efficacy of this two-step approach in a tumor efficacy model. Based on published results from murine cancer models, a tissue concentration of ~20 μg/g is sufficient to eliminate all detectable tumor^28^, and this amount will serve as a target for our optimization studies. Nevertheless, this initial design serves as a robust proof-of-concept with a greater than 5-fold increase in tumor tissue concentrations of Pax compared to the administration of Pax alone. Additionally, a 2-step system may clarify and simplify the FDA regulatory process (i.e., 2-step delivery—a ‘device’ with the potential for 510k approval used in concert with standard systemic delivery of a chemotherapeutic versus single component delivery—‘drug-device’ combination product) and, therefore, quicken the time to the Clinic.

While the proposed mechanism of action of this system consists of tumoral uptake of eNPs followed by intra-endosomal swelling and subsequent accumulation of Pax (Fig. 1), other mechanisms are possible. For example, eNPs may swell in the mildly acidic extracellular environment of the tumor (~pH 6–6.5) and, without ever entering the cell, concentrate drug into the tumoral environment. Unfortunately, the *in vivo* quantification of Pax concentrations in the tumor does not provide clarity on this point as it is a bulk measurement.

The idea of using a “two-step” delivery system originated with antibody-mediated localization of radioimmunotherapy to hematopoietic cancers^29^,^30^,^31^,^32^,^33^,^34^,^35^. In these studies by Press *et al.*, a “targeting vehicle”, such as a streptavidin labeled antibody, is administered and binds tightly to target cells due to the antibody-cell receptor interaction. Radiolabeled-biotin conjugate is subsequently administered, binding strongly to the streptavidin-antibody conjugate due to the high biotin-streptavidin affinity^36^, and thus localizes the radiotherapy to the target cells *in vivo* while any unbound radiolabeled-biotin is rapidly cleared from the systemic circulation. Alternative approaches to “pre-targeted” radioimmunotherapy have also been described using bi-specific monovalent antibody-antihapten conjugates with similar results^30^,^34^. However, suboptimal performance of streptavidin-biotin systems *in vivo* occurs due to the inability to effectively eliminate biotin from patient diets^37^.

Corresponding two-step, biotin-streptavidin NP-based systems have also been developed. Chan *et al.* directly extended the above two-step concept by demonstrating that injection of a biotin-labeled NP, which localizes to tumors, followed by a second injection of a streptavidin-labeled contrast agent resulted in rapid accumulation of the contrast agent within the NP/tumor^37^.

Other groups have extended the two-step concept still further from biotin-streptavidin to one NP followed by a second NP formulation (i.e., NP/NP). Bhatia *et al.* used an injection of gold-nanorods to heat tumors (via plasmonic resonance), thereby “priming” the tissue for accumulation of a subsequently injected drug-loaded, targeted liposome^38^,^39^,^40^. Similarly, Nel *et al.* showed sequential delivery of peptide-loaded silica NPs that disrupt the pericyte coverage around tumoral blood vessels increased subsequent accumulation of gemcitabine-loaded NPs to the tumor via increased permeability of the tumor vasculature^41^.

The current manuscript distinguishes itself from these previous two-step models in three important ways. First, unlike published NP/small molecule systems that require a high-affinity antibody-based attraction between the NP and small molecule (i.e., biotin-streptavidin), the current system leverages non-specific, non-targeted hydrophobic partitioning of Pax into the eNP to achieve accumulation. Second, in contrast to two-step NP/NP systems, this system uses a NP/small molecule approach. And, third, unlike NP/NP systems that depend upon either: extrinsic activation (e.g., magnetic radiation) or tumor-specific targeting proteins/antibodies to assist in localization to tumor of at least one of the two components, the eNP/Pax system requires no extrinsic activation or antibody targeting. One limitation of the current study is that, while the work by Press *et al.* was able to circumvent toxicities associated with traditional (single-step) delivery, the parameters of drug- and nanoparticle-dosing in this study were selected to be within a well-tolerated range; therefore, no toxicity or difference in toxicity between Pax alone an eNP + Pax was discernable. Specifically, the dose of paclitaxel was 10 mg/kg (selected based upon previously published animal models^12^,^27^); this dose is non-toxic and does not result in measureable side effects. This dose also enabled us to conduct the study while minimizing other factors (i.e., treatment-related morbidity/mortality) that may have complicated the analysis.

Lastly, a recent publication by Brudno *et al.* described the concept of a refillable drug depot based on the sequence specificity and strength of binding of oligodeoxynucleotides (ODNs)^42^. An intratumoral injection of alginate polymers conjugated to ODNs followed by a subsequent injection of ODN-bound drug was found to sequester drug within the tumor. The authors relied on the enhanced retention and permeability effect to bring the two components of their system close enough for the high specificity and affinity of the ODN-based interactions to take effect. Using this two-step approach they estimated that ~1% of the injected dose of drug accumulated in the target tissue. The results of the current study are encouraging as they demonstrate that a system based on non-specific interactions can achieve similar effects (i.e., accumulation of ~0.5% of the injected dose in the target tissue) to a highly-engineered, tuned, and specific ODN-based system.

The current study demonstrates a partition-coefficient driven approach to drug delivery and presents a new strategy for NP drug delivery wherein the component traditionally labeled as the “carrier” (i.e., eNP) is administered separately from its “payload” and subsequently concentrates the systemically administered payload to a target tissue. Both the *in vitro* and *in vivo* results show a local increase in Pax concentration of 4–5X and this ability is not common among all nanoparticles (e.g., as seen by the lack of drug concentration with PLGA-NPs). The result that Pax-eNPs are more efficient at tumoral delivery than eNPs + Pax is neither surprising nor the main focus of this manuscript; rather, the focus is on the surprising and unprecedented result that by pre-treating with an untargeted nanoparticle system, a similarly untargeted drug can be concentrated into a specific tissue (i.e., tumor) at significantly higher levels than is achieved compared to drug-alone treatments. This approach represents a significant change from the current clinical regimen of a systemic bolus delivery of chemotherapy and also from the highly researched paradigm of nanoparticle-mediated (i.e., encapsulated or covalently bound) drug delivery. This method provides a means to achieve a superior concentration of chemotherapy within the tumor and prolonged intra-tumoral drug exposure for a minimum of several days. Continued development and evaluation of new functional materials and delivery approaches will provide further insight into and broaden the field of nanotechnology for drug delivery.

## Methods

### General Procedures and Materials

Nanoparticle formulations used in this study were prepared using a two-step mini-emulsion and base-catalyzed polymerization according to previously published procedures^10^,^11^. For *in vitro* studies: complete growth media refers to RPMI supplemented with 10% fetal bovine serum (FBS) and 1% glutamine-penicillin-streptomycin (GPS). Phosphate buffered saline (PBS) was used without calcium and magnesium supplementation unless otherwise noted. All cells were cultured at 37 °C with 5% CO_2_ atmosphere. Media was replaced every other day during culture unless otherwise specified. All cell lines were used before passage 15.

### Quantification of paclitaxel partitioning in nanoparticles

To quantify the partitioning of paclitaxel into nanoparticles, an aliquot of 150 uL of particles (either eNPs or succinic acid-based eNPs) was diluted in 6 mL of 10 mM pH 5.0 acetate buffer and added to a dialysis cassette (10,000 molecular weight cut off). This was, in turn, suspended in 50 mL of a solution of 2 ug/mL paclitaxel solubilized by the addition of 0.3% wt/vol sodium dodecyl sulfate (i.e., aqueous sink). Aliquots of both the eNP and sink were taken at time 0 and after 24 hours. Paclitaxel concentrations were measured via reverse-phase high performance liquid chromatography using a 70:30 acetonitrile:water mobile phase (flow rate 0.5 mL/min) and a Hamilton C18 column (injection volume 20 uL).

### *In vitro* quantification of Oregon Green-labeled paclitaxel (green-Pax) concentration into eNP pre-treated cells

To quantify the intracellular drug concentrating capability of expansile nanoparticles (eNPs) after two days of incubation with cells, MSTO-211H cells were plated in 96-well plates at a density of 2,000 cells/well and allowed to adhere for 24 hr. The media was then removed and the cells were incubated for an additional 48 hr with 2 mL media containing 50 μg/mL (polymer concentration) eNPs, PLGA-NPs or with media alone. The media was then aspirated and the cells were washed 2X with 2 mL PBS, and incubated for 4 hr with media containing 25 μg/mL green-Pax. After 4 hr, the media was aspirated and the cells were washed 2X with 1 mL cold PBS followed by lysing with 100 μL Promega Lysis Buffer 1X. A fluorescence plate reader (Molecular Devices Spectra Max M5) was then used to measure fluorescence (λ_ex_ = 488 nm; λ_em_ = 518 nm; cutoff filter 515 nm). All measurements were performed in at least triplicate.

### Fluorescence microscopy of green-Pax concentration into eNP pre-treated cells

To confirm the intracellular drug concentrating capability of eNPs after two days of incubation with cells, we measured uptake of green-Pax in eNP pre-treated MSTO-211H human mesothelioma cancer cells. MSTO-211H cells were plated in 6-well plates at a density of 200,000 cells/well and allowed to adhere for 24 hr on #1.5 18 × 18 mm glass coverslips. The media was then removed and the cells were incubated for an additional 48 hr with 2 mL media containing 50 μg/mL (polymer concentration) eNPs or with media alone. The media was then aspirated and the cells were washed 2X with 2 mL PBS, and incubated for 4 hr with media containing 25 μg/mL green-Pax. After 4 hr, the media was aspirated and the cells were washed 2X with 1 mL cold PBS, fixed in 3.7% formaldehyde for 20 minutes, and washed an additional 2X with 1 mL PBS and 2X with 1 mL PBS containing calcium and magnesium, then stained with 3 μg/mL Hoechst trihydrochloride trihydrate (nuclear stain) and 100 μg/mL Concanavalin A 633 conjugate (membrane stain) for 8 minutes. The cells were washed again 2X with 1 mL PBS containing calcium and magnesium and the coverslips then mounted on glass slides with Prolong Gold Anti-Fade reagent. Coverslips were imaged using a Zeiss LSM 510 meta inverted confocal microscope.

### *In vivo* characterization of drug concentration via eNP pre-treatment

Animal experiments were approved by the Institutional Animal Care and Use Committee and conducted in strict compliance with all federal and institutional guidelines. Intraperitoneal (IP) mesothelioma was established by IP injection of 5 × 10^6^ MSTO-211H-luciferase transfected cells in female, athymic, nude, 6–8 week old mice (Nu/J, Jackson Laboratory, Bar Harbor, ME). On day 11 following tumor cell injections, mice were randomized into two groups to receive IP injections of 0.3 mL of 20 μg/mL empty blue-eNPs or saline. Two days later (day 13), all mice received IP injections of 10 mg/kg green-Pax-C/E in saline (6 mg/mL stock green-Pax-C/E diluted to 1 mg/mL with saline). Three days later, on day 16, all mice were euthanized. Upon sacrifice, the IP space was opened and examined using UVA 365 nm and UVC 254 nm light from a hand-held Wood’s lamp. Tumor tissues were harvested, fixed, stained with propidium iodide (nucleus) and imaged using an Olympus FluoView 1000 scanning confocal laser microscope using DAPI, FITC, and RHO filters to isolate the blue-eNP, green-Pax, and nuclear signals, respectively.

### *In vivo* quantification of drug concentration via eNP pre-treatment

Animal experiments were performed exactly as above but with un-labeled Pax and eNPs instead of green-Pax and blue-eNPs. IP mesothelioma was established by IP injection of 5 × 10^6^ MSTO-211H-luciferase transfected cells in female, athymic, nude, 6–8 week old mice. On day 11 following tumor cell injections, mice were randomized into three groups to receive IP injections of 0.3 mL of 20 μg/mL empty eNPs, equivalent polymer weight of PLGA-NPs, or saline. Two days later (day 13), all mice received IP injections of 10 mg/kg Pax-C/E in saline (6 mg/mL stock Pax-C/E diluted to 1 mg/mL with saline). Three days later, on day 16, all mice were euthanized. Upon sacrifice, all visible tumors in the IP space were dissected, harvested, weighed, then snap frozen, and stored at −80 °C for Pax quantification.

Liquid chromatography-triple quadrapole mass spectrometry (LC/MS/MS) (Apredica Inc., Watertown, MA) was used to measure Pax concentrations within the tumor tissues. Pax was extracted from the tissue with three volumes of methanol containing an internal standard to validate the extraction procedure. This was centrifuged for 5 min at 14,000 RPM to remove precipitated protein. The supernatant was collected and analyzed by LC/MS/MS using an Agilent 6410 mass spectrometer coupled with an Agilent 1200 HPLC and a CTC PAL chilled autosampler, controlled by MassHunter software (Agilent). After separation on a C18 reverse phase HPLC column (Agilent, Waters, or equivalent) using an acetonitrile-water gradient system, peaks were analyzed by mass spectrometry (MS) using ESI ionization in MRM mode. The limit of detection was 40 ng/g.

## Additional Information

**How to cite this article**: Colby, A. H. *et al.* Two-Step Delivery: Exploiting the Partition Coefficient Concept to Increase Intratumoral Paclitaxel Concentrations *In vivo* Using Responsive Nanoparticles. *Sci. Rep.* **6**, 18720; doi: 10.1038/srep18720 (2016).
